# What do youth need to know about puberty? A scoping review protocol to identify puberty education competencies

**DOI:** 10.1371/journal.pone.0351147

**Published:** 2026-06-09

**Authors:** Marie A. Brault, Nanki Singh, Nikita Kakkad, Melissa Peskin, Anthony Betori, Gregory Laynor, Emily Naiser

**Affiliations:** 1 Institute for Excellence in Health Equity, Section for Global Health, New York University Grossman School of Medicine, New York, New York, United States of America; 2 Department of Population Health, New York University Grossman School of Medicine, New York, New York, United States of America; 3 New York University Grossman School of Medicine, New York, New York, United States of America; 4 Department of Health Promotion and Behavioral Sciences, The University of Texas Health Science Center at Houston School of Public Health, Houston, Texas, United States of America; 5 Healthy Futures of Texas, San Antonio, Texas, United States of America; 6 Health Sciences Library, New York University Grossman School of Medicine, New York, New York, United States of America; 7 Public Policy Research Institute, Texas A&M University, College Station, Texas, United States of America; KU Leuven University Hospitals Leuven: Katholieke Universiteit Leuven Universitaire Ziekenhuizen Leuven, BELGIUM

## Abstract

**Introduction:**

Puberty is a key transition point in adolescents’ lives that plays a foundational role in shaping health behaviors and outcomes across one’s life course. This period holds significant potential to empower adolescents and support autonomy in health and well-being, but limited puberty education curricula exist for early adolescents (age 8–14), and those that do exist vary in content. There is a paucity of evaluations of puberty competencies and limited consensus on what competencies should be measured to assess effectiveness or even how to measure these competencies.

**Objective:**

The objective of this scoping review is to systematically map and characterize the outcomes, domains, and instruments used to evaluate puberty education curricula for early adolescents aged 8–14 years. In accordance with PRISMA-ScR and JBI scoping review guidance, this review does not synthesize effect sizes or assess intervention efficacy, but maps the breadth of evidence to identify conceptual gaps and inform future framework development.

**Methods:**

The review protocol is registered with the Open Science Framework (OSF). We will search PubMed, CINAHL, PsycInfo, ERIC, Education Source, Scopus, Web of Science Core Collection, ProQuest Dissertations and Theses Global, and OpenAlex for relevant sources. Two reviewers will independently screen and extract studies that meet inclusion criteria using our data extraction tool.

**Expected outputs:**

Findings from the scoping review will be synthesized to create an overarching framework that can guide approaches to the development and evaluation of puberty curricula targeted to early adolescents. Focus group discussions with adolescents, parents, and school representatives will be conducted to assess the applicability and appropriateness of identified competencies and evaluation measures prior to broader dissemination. Insights from this scoping review will ultimately be used to inform the implementation and evaluation of puberty education.

## Introduction

Puberty marks a pivotal developmental transition defined by profound biological, psychological, and social changes. This period signals the onset of adolescence and plays a foundational role in shaping individuals’ physical health, emotional well-being, cognitive development, and socio-economic trajectories across the life course. [1] The developmental phase of puberty thus represents a critical window of opportunity to engage young people in ways that support their transition to adulthood, laying the groundwork for long-term health, well-being, and social integration [2,3].

Despite its universality, the experience of puberty is frequently complicated by layers of misinformation, reticence from adults, stigma regarding changing bodies, and culturally-embedded taboos. [4–7] These sociocultural constraints inhibit open discourse and contribute to gaps in access to accurate, age-appropriate, and contextually relevant information for adolescents. [8,9] These barriers are not uniformly distributed; adolescents from low- and middle-income countries, racial and ethnic minority groups, and lower socioeconomic backgrounds face compounded disadvantages in accessing accurate puberty information, reflecting broader structural inequities in health education. [10,11] Further, inadequate puberty education has been associated with confusion, heightened anxiety, and suboptimal health-seeking behaviors, as adolescents navigate these transitions with limited guidance. [12–14] Emerging evidence underscores the imperative for a comprehensive, developmentally appropriate approach to puberty education—one that extends beyond biological instruction to encompass emotional regulation, body image, cultural norms, interpersonal relationships, and informed decision-making. [15].

Early adolescence—typically defined as the period between 8 and 14 years of age—represents a critical yet persistently under-addressed stage in sexual and reproductive health (SRH) education. Marked by rapid physical, emotional, and social transitions, this developmental phase is often neglected in global and national curricula, which tend to prioritize older adolescents (15 years and above). [16–19] This omission is particularly concerning given that early adolescence is a formative period during which foundational attitudes and behaviors related to identity, body image, sexuality, and mental health emerge. [20,21] More developmentally appropriate and age-specific programming is needed to support this population. Early adolescence is also when dominant social scripts surrounding bodies, identities, and relationships are first internalized, shaping lifelong patterns of self-concept and social interaction. [22,23]

Despite growing recognition of puberty as a critical developmental window, puberty education remains highly fragmented in content and implementation. [2,24] In the United States, the extent to which schools offer structured puberty education is poorly documented, and the content delivered varies substantially in terms of scope, duration, and pedagogical approach. [25] Many curricula continue to center menstruation education while neglecting the distinct experiences and needs of boys. [25–32] These omissions reflect assumptions about whose bodies are seen as appropriate subjects of education and which developmental experiences are deemed worthy of institutional attention. [33] As such, puberty curricula transmit knowledge, and reproduce normative constructions of identity, embodiment, and developmental trajectories. [34,35] Sex-segregated approaches may hinder adolescents’ understanding of peer experiences, exclude critical content, and reduce youth engagement by presenting material that feels irrelevant or exclusionary. [29,36,37]

Equally important—yet underexplored—are the conceptual and methodological challenges associated with evaluating the effectiveness of puberty education. In contrast to sexual health education for older youth, which often relies on assessing established behavioral proxies/competencies such as condom use or delayed sexual initiation, there is limited consensus on what constitutes a “competent” or developmentally appropriate understanding of puberty. While many programs report improvements in factual knowledge, few assess shifts in emotional regulation, body image or literacy, or the evolving sense of self. [31,38] The lack of standardized, theory-informed outcome measures hinders efforts to determine whether puberty education is meeting its intended objectives. A recent systematic review highlights the scarcity of rigorous evaluations and notes that existing assessments are often methodologically inconsistent, relying on tools that may fail to capture the nuanced and longitudinal nature of developmental change. [25,32,39] In the absence of validated metrics, it remains difficult to assess impact, compare interventions, or iteratively refine program design.

National and global frameworks for comprehensive sexuality education define comprehensive sexuality education as a curriculum-based process of teaching and learning about the cognitive, emotional, physical, and social aspects of sexuality. [15] Within these frameworks, puberty education is identified as a foundational component of adolescent development and health education. [15,27,40] In the United States, the National Sex Education Standards (NSES) are considered the gold standard for comprehensive sexuality education. Many states and some cities also have sex education standards, which incorporate puberty to varying degrees and with varying levels of alignment with NSES. [41] While international and national guidelines are increasingly adopted in policy and programmatic settings, there remains limited understanding of how, and to what extent, youth internalize and apply puberty-related competencies in practice.

A central limitation in current approaches to puberty education is the focus on cognitive learning outcomes—typically defined as the acquisition and recall of information, such as knowledge of reproductive anatomy or the stages of pubertal development. While such assessment strategies may be suitable for older youth, younger adolescents require pedagogical methods that are age-appropriate, participatory, and emotionally resonant to meaningfully engage with content. [42,43]

Compounding this challenge is the lack of consensus around which competencies early adolescents should be expected to acquire through puberty education. Although the NSES offer specific grade-level learning objectives, they provide limited guidance on how to assess psychosocial competencies. Clarifying which competencies are foundational for navigating puberty successfully—beyond basic knowledge—remains unresolved. In the absence of clearly articulated developmental benchmarks and validated assessment tools, efforts to standardize puberty education and rigorously evaluate its effectiveness across diverse sociocultural contexts remain constrained. [3]

There is a pressing need for a comprehensive synthesis of the current evidence base. In this context, a scoping review is a methodologically appropriate approach to systematically map the breadth and scope of available literature, clarify conceptual boundaries, and identify critical gaps in knowledge and practice within this evolving field. [44,45] A scoping review is the most appropriate methodology for this purpose. Unlike systematic reviews, which synthesize effect sizes and assess intervention efficacy, scoping reviews map the extent, range, and nature of available evidence, identify conceptual gaps, and clarify definitional boundaries — without appraising the quality of individual studies. This distinction is critical: the present review does not evaluate the rigor of puberty education evaluations but rather maps and categorizes the tools and competency frameworks they employ. [45–47]

This scoping review aims to identify and synthesize the core competencies that young people require to navigate puberty effectively. These competencies span multiple, interrelated domains, including biological literacy (e.g., knowledge of menstruation, spermarche, and hormonal changes), body and self-care practices (e.g., hygiene management, menstrual product use), emotional and psychological self-awareness, identity development, interpersonal communication, and the ability to identify and engage trusted support networks and health services. [20,40,48] Competency, as used in this review, is defined as an integrated combination of knowledge, skills, and attitudes that enables individuals to perform tasks and navigate challenges in context-appropriate ways. [10,49] This formulation extends beyond the recall of factual information to encompass emotional regulation, behavioral application, and attitudinal change—dimensions increasingly recognized as central to health literacy and adolescent health education. [50,51] For the purpose of this review, puberty-related competenceis are understood as the knowledge, emotional regulation skills, and learning objectives that equip adolescents to understand, manage, and respond to the physical and psychosocial changes of puberty. [52,53] Part of the contribution of this review is to clarify and operationalize these competency domains as they appear across existing evaluation tools,

Articulating these competencies is especially urgent in the context of current global and structural challenges. The COVID-19 pandemic disrupted access to school-based sexuality education for millions of adolescents worldwide, exacerbating pre-existing unavailability and amplifying the need for community-based and digital delivery models. [54] Concurrently, growing attention to student-centered approaches, adolescent well-being, and evidence-based health education reinforces the need for puberty education that supports healthy youth development. [55,56]

Although one prior review addressed puberty education more broadly, it underscored a lack of rigorous evaluation and evidence-based practice within the field. [25] Other studies examining school-based or community-delivered sexuality education tend to exclude detailed evaluations or focus primarily on broader sexual and reproductive health (SRH) curricula targeted at older adolescents. Across these studies, there is high heterogeneity in intervention content and evaluation methodologies, limiting the comparability of outcomes across contexts. [57–59]

This review is guided by the following research questions: (1) What tools and instruments have been used to evaluate puberty education curricula targeting adolescents aged 8–14 years? (2) What competency domains — spanning knowledge, skills, attitudes, and behaviors — do these tools measure? (3) What are the methodological characteristics of these evaluations?

This scoping review will identify and critically analyze existing tools used to evaluate puberty education curricula and assess puberty-related competencies, with the overarching aim of mapping current strategies and laying the foundation for a theory-informed, developmentally appropriate assessment framework. A preliminary search of MEDLINE (PubMed), Google Scholar, the Open Science Framework (OSF), and PROSPERO revealed no existing or planned reviews focused specifically on evaluation tools for puberty education in adolescents aged 8–14 years. This review will adhere to the JBI methodological framework for scoping reviews and will be reported in accordance with the Preferred Reporting Items for Systematic Reviews and Meta-Analyses extension for Scoping Reviews. By systematically mapping the tools, outcomes, and competency domains assessed across this literature, this review will contribute an evidence-based assessment framework to inform future research, curricular development, and educational practice across global settings.

### Objective

The primary objective of this scoping review is to identify and synthesize evidence related to the evaluation of puberty education curricula for early adolescents, with a specific focus on the outcomes assessed, the domains and competencies measured, and the validity of the instruments used. To achieve this, the review will pursue four interrelated aims: (1) to identify and characterize the assessment tools used to evaluate the effectiveness of puberty education curricula targeting pre-teens and early adolescents in school and community settings; (2) to examine the specific competencies, knowledge domains, and psychosocial issues addressed by these tools; (3) to document the demographic and contextual characteristics of the youth populations included in these interventions and evaluations; and (4) to determine whether the identified tools and outcome measures have undergone psychometric validation, particularly with respect to applicability across diverse adolescent populations.

Recognizing the global relevance of puberty education, this review will adopt a broad lens on implementation settings, encompassing formal educational institutions informal, community-based platforms, and digital platforms. By doing so, the review aims to generate a comprehensive understanding of how puberty curricula are evaluated across contexts and to inform the development of standardized, contextually grounded, and developmentally appropriate assessment frameworks.

## Eligibility criteria

### Rationale for criteria selection

Participant age between 8–14 years was selected based on medical and social literature defining the ages at which youth are biologically and psychosocially initiating puberty. [60,61] Given that the focus of this scoping review is to understand and synthesize tools used to evaluate puberty curricula rather than to synthesize the curricula itself, quality measures and mode of delivery of the curricula are not included in inclusion criteria.

Studies originally written in non-English languages that do not have an English-translated copy already available will be translated with the use of AI-assisted translation software with review by translation personnel at one of the co-author’s universities (the authorship team represents three institutions) to ensure accuracy. Grey literature is being included as it provides an important source of potential evaluation tools, and grey literature will undergo the same screening process as peer-reviewed literature included.

Consistent with scoping review methodology, formal risk-of-bias appraisal of included studies will not be conducted. However, the psychometric properties of evaluation tools (e.g., reliability, validity, responsiveness) will be extracted and reported descriptively where available, given the review’s specific focus on assessment instruments. Grey literature will undergo the same dual-reviewer screening process as peer-reviewed literature; where psychometric data are absent, this will be noted as a finding rather than a basis for exclusion.

**Table:** 

	*Inclusion*	*Exclusion*
Participants	i) Studies where the primary target population is youth aged 8–14 years ii) Studies with mixed age ranges where data for youth aged 8–14 can be extracted or disaggregated separately	i) Studies where all participants are younger than 8 or older than 14 years and/or age-specific data for the 8–14 range cannot be extracted or disaggregated
Concept	i) Puberty education curricula or sexual and reproductive health curricula with puberty-specific outcomes that have undergone formal evaluation ii) Curricula delivered via any modality, including classroom-based, community-based, and technology-mediated formats (e.g., web-based platforms, mobile applications, digital games) iii) Evaluations using quantitative, qualitative, or mixed-methods approaches iv) Outcome measures spanning knowledge, attitudes, skills, behaviors, and social-emotional domains, whether validated or not	i) Curricula with no puberty-specific content or outcomes ii) Curricula that have not undergone any form of evaluation iii) Interventions where puberty education is incidental rather than a primary or stated component
Context and Delivery Mode	i) School or community-based educational settings for youth ii) Digital delivery iii) United States and global studies	i) Clinic-based or healthcare facility educational interventions
Type of study/ Source	i) Peer-reviewed primary studies and reviews reporting evaluation of puberty education curricula ii) Federally funded curricula reviewed through the Teen Pregnancy Prevention Program (TPP) or equivalent national programs, sourced systematically from program repositories iii) Dissertations and theses from ProQuest Dissertations & Theses Global iv) Programmatic reports and grey literature where evaluation methods and outcomes are clearly described; all grey literature will undergo the same dual-reviewer screening process as peer-reviewed literature	i) Commentaries, editorials, opinion pieces, or theoretical papers without empirical evaluation data i i) Curricula documents with no associated evaluation data iii) Grey literature where methods and outcomes are insufficiently described to determine eligibility

## Methods

### Search strategy

A core search strategy was designed in PubMed to comprehensively identify studies at the intersection of three core concepts of the research question: terms related to puberty; terms related to curriculum or education; terms related to the implementation and evaluation or programs and competency outcomes. Each set of terms include free-text keywords and structured vocabulary (MeSH terms) when available for the concept. The terms within each set are combined with the Boolean operator OR and the three sets are joined with the Boolean operator AND.

(“pubert*” [tiab] OR “puberty” [mesh]) OR “menstruation” [mesh] OR “menstruat*” [tiab]) **AND** (“curricul*” [tiab] OR “curriculum” [mesh] OR “educat*” [tiab] OR “education” [subheading] OR “health education” [mesh] OR “sex education” [mesh]) **AND** (“attitude*” [tiab] OR “behavior” [mesh] OR “behavior*” [tiab] OR “competen*” [tiab] OR “evaluat*” [tiab] OR “health knowledge, attitudes, practice” [mesh] OR “implement*” [tiab] OR “implementation science” [mesh] OR “knowledge” [mesh] OR “knowledge*” [tiab] OR “measure*” [tiab] OR “pilot projects” [mesh] OR “program evaluation” [mesh] OR “puberty/psychology” [mesh]) **AND** english[Language] **AND** (“2004”[Date – Publication]: “3000”[Date – Publication])

The search strategy will be translated to databases encompassing the range of disciplines related to the research question as well as cross-disciplinary databases: CINAHL, PsycInfo, ERIC, Education Source, Scopus, Web of Science Core Collection, ProQuest Dissertations and Theses Global, and OpenAlex. Each translation will maintain conceptual consistency from the core search strategy, utilizing database-specific structured vocabulary when available.

Search results will be imported from each database and imported to Covidence.

Databases included in the search strategy include some grey literature sources, such as dissertations. Additional potentially relevant grey literature sources will be identified within included studies identified from the initial database searches. Additionally, grey literature sources such as the Teen Pregnancy Prevention Program repository and sources from ProQuest Dissertations and Theses Global will be searched systematically using predefined search terms in line with the search strategy described above. Grey literature sources referred to in identified peer-reviewed studies will be systematically reviewed by multiple study team members to ensure that appropriate information is being extracted for full screening and review.

To ensure the search is as comprehensive and up to date as possible, the CitationChaser tool will be utilized to identify any additional studies citing or cited by studies included in the review from the initial search [62].

### Scoping review timeline

The scoping review protocol was pre-registered with the Open Science Framework (OSF) in September 2025 and subsequently submitted to *Plos One*. Depending on the peer-review timeline, we plan to run the search strategy in May or June 2026, followed by title/abstract screening in July and August 2026. We aim to commence full study screening and finalize the data extraction form in September 2026. Data extraction and data cleaning will occur in October and November of 2026. We anticipate that all data will be collected by early December 2026, with data analysis completed by the end of January 2027, and the manuscript containing review findings will be prepared after this date. We can confirm that no data has been collected or analyzed at this time, and no date will be collected or analyzed until the protocol has been accepted for publication.

### Evidence selection

Screening of studies will occur in two phases utilizing the Covidence platform: screening of titles/abstracts, followed by a second phase of full text screening. All titles/abstracts retrieved from the deduplicated database searches will be screened independently by two reviewers based on the inclusion/exclusion criteria established in the protocol. Titles/abstracts that meet criteria, or titles/abstract that lack adequate information to evaluate eligibility, will move forward to full-text screening. Any discrepancies in title/abstract screening will be adjudicated by a third reviewer. Two reviewers will independently make full text screening decisions; and any discrepancies will again be adjudicated by a third reviewer.

The review team will periodically monitor interrater reliability using the kappa statistic generated by Covidence. [63] If the kappa is less than.60, the team will review the eligibility criteria together to ensure the team is aligned in interpreting and applying the criteria and if any clarifications or refinements of the criteria are needed.

### Data extraction

A data extraction form (see S1 File for extraction form in the supporting information) will first be piloted by the review team on a sample of studies that meet eligibility criteria for inclusion in the review, and are reflective of the types of data available. The research team completed preliminary refinement of the types of outcomes assessed through literature searching and discussion. Preliminary categories for extraction include:

- Study Participants (age, grade level, other demographic information reported)
- Study Context (study setting, language of instruction, curriculum facilitators)
- Study Design (methods for assessment, description of assessment tool(s)/instrument(s), measures of instrument validity and/or reliability)
- Types of outcomes assessed (behaviors, attitudes, information, practices, social-emotional well-being, others)
- Summary of author’s reported findings
- Reviewer’s summary of how findings related to review questions

The review team will refine the data extraction form based on discussion during the pilot phase of extraction.

Data extraction will be conducted in Covidence on all studies included after full text screening. Two reviewers will independently conduct data extraction for each study; a third reviewer will adjudicate any data extraction discrepancies. Final data extraction will be exported in CSV format for data analysis and presentation.

### Data analysis and presentation

Descriptive statistics will be used to describe study participant demographics across the literature and frequency of the study settings, study designs, and types of outcomes found in the literature. Given that inclusion criteria for this review includes age, a description of how ages included in identified puberty curricula and rationale for choosing age ranges will be included in our analysis. Where reported by study authors, the psychometric properties of evaluation tools will be extracted and described, including internal consistency (e.g., Cronbach’s alpha), test-retest reliability, construct and content validity, and responsiveness to change. Absence of psychometric data will be noted as a descriptive finding; consistent with scoping review methodology, this will not serve as a basis for exclusion. [47]

A qualitative content analysis will be conducted to address the review’s research questions, following the JBI methodological framework for scoping reviews and reported in accordance with PRISMA-ScR. [45,46,64] Analysis will focus on mapping and categorizing the types of tools and outcome measures used to evaluate puberty curricula, without appraising the effectiveness of individual interventions or the methodological rigor of their evaluations. As part of this, we will compare how authors define and operationalize puberty-related outcomes. Specifically, we will systematically compare how outcome measures in each study are framed — as knowledge outcomes, attitudinal outcomes, behavioral outcomes, skills-based competencies, or social-emotional learning objectives — and assess the degree to which these map onto our a priori definition of competency as an integrated combination of knowledge, skills, and attitudes. Where the data support it, visual synthesis methods will be used to present findings, including a competency mapping framework illustrating how puberty-related outcomes cluster across knowledge, attitudinal, behavioral, and skills-based domains, and a conceptual model situating puberty competencies within broader adolescent developmental and health outcomes frameworks. Additionally, given the sensitivity and cultural differences that influence perceptions and experience of puberty, secondary analysis will contextualize the findings by characterizing outcomes and assessment measures by demographic and socio-economic backgrounds. We will also seek to develop a repository of tools to assess puberty outcomes.

## Discussion

This scoping review aims to synthesize the growing body of literature that acknowledges early adolescence and puberty as a critical time in psychosocial development, yet one unevenly addressed due to heterogenous approaches in educational and public health programming. While national and international guidelines exist to guide puberty education, they remain focused on sexual and reproductive health competencies, are often tailored to an older adolescent demographic, and are hindered by a lack of established goal competencies and assessment measures.

An overview of what puberty educational content is being delivered and methods used to evaluate the effectiveness of puberty education will lay the foundation for future, evidence-based puberty education interventions. Mapping the frequency and type of evaluation tools used, and highlighting gaps in age- and culturally-appropriate pedagogy may be used to guide future studies that address developmental needs and educational inequities from the outset. Ultimately, this work aims to provide a starting point for a contextually-responsive, comprehensive approach to puberty education that affirms adolescents with all lived experiences, and a path forward for reshaping how puberty education for early adolescents is delivered and measured, both locally and globally.

### Limitations

While our search strategy was developed to be comprehensive and systematic answering our research questions, it is possible that relevant evaluations or programs will not be captured if not published in peer-reviewed literature or indexed in major databases. Given that there is a wide variety in which competencies are defined as important and how competencies are measured, it may be difficult to draw comparisons between studies that significantly differ in methodological approaches.

In addition, comparing studies across global contexts presents limitations due to variance in cultural norms, education delivery, and infrastructure. Differences in how puberty is conceptualized across regions present an added layer of heterogeneity in prioritized puberty competencies and evaluation metrics. While these differences are important for culturally sensitive programming, synthesizing information across varied contexts may lead to oversimplification. Furthermore, language barriers and inconsistent reporting standards may limit the inclusion of non-English or non-Western studies. These contextual differences may limit the generalizability of the findings, or risk losing nuance in individual approaches. To mitigate this, we will conduct qualitative data abstraction and thematic analysis of included studies, allowing us to identify context-specific insights.

### Dissemination plan

Findings from this scoping review will be disseminated through multiple complementary channels targeting research, policy, and practice audiences. A peer-reviewed manuscript will be submitted to a journal specializing in adolescent health, sexual and reproductive health, or health education. In parallel, plain-language policy briefs will be developed for health education specialists, curriculum developers, school systems, and public health practitioners to support awareness of the current evidence landscape in puberty education programming.

Findings will additionally be presented at national and international academic conferences in adolescent health and sexual and reproductive health. All dissemination materials will be made openly accessible where possible, in accordance with open science principles, to maximise reach across resource-diverse settings.

As a knowledge translation activity subsequent to — and informed by — this review, findings will be subject to participatory validation through structured focus group discussions with adolescents, caregivers, and school-based education stakeholders. These discussions will assess the relevance, acceptability, and perceived applicability of identified competency domains and evaluation approaches across diverse youth populations. Insights generated through this process will inform the iterative refinement of the assessment framework and support its application to the development and evaluation of puberty education curricula, including programs currently under development for early adolescent populations. [59]

Together, this dissemination strategy aims to make the findings of this review accessible to research, policy, and practice audiences, providing a foundational evidence map and replicable assessment framework to support future curriculum evaluation, programme development, and implementation research in puberty education across global settings.

## Supporting information

S1 File Article data extraction form. This form will be used to collect information as reported by authors in the articles that meet inclusion and exclusion criteria.
(DOCX)

S2 File Completed PRISMA-P checklist for planning, development, and reporting of systematic review protocols. (DOCX)
