# Supplementary material for: What do youth need to know about puberty? A scoping review protocol to identify puberty education competencies
Source: PLoS One. 2026 Jun 9;21(6):e0351147. doi: 10.1371/journal.pone.0351147 (PMC13249200; doi:10.1371/journal.pone.0351147)
Supplement: S1 File — This form will be used to collect information as reported by authors in the articles that meet inclusion and exclusion criteria. (DOCX) [file pone.0351147.s001.docx]

Draft of Extraction Form for Scoping Review

Note: This form will be entered into REDCap to ease data entry and management.

1. Full citation

____________________________________________________________________

____________________________________________________________________

____________________________________________________________________

1. Year of publication ________________
2. Name of curriculum, if provided ___________________________________________
3. Brief description of curriculum being evaluated (as described in article)

____________________________________________________________________

____________________________________________________________________

____________________________________________________________________

1. Ages of youth participants who received the curriculum (Select all that apply):
   - 8
   - 9
   - 10
   - 11
   - 12
   - 13
   - 14
   - 15+
   - Other description of age provided in article ______________________
   - Unsure (not clearly stated in article)
2. Demographics of youth participants (enter characteristics of youth race, ethnicity, sex, or other characteristics as reported in the manuscript) ____________________________________________________________________
3. In what language, was the curriculum provided to the youth? (select all that apply)
   - English
   - Spanish
   - Other _______________________________
4. In what setting was the curriculum provided? (select all that apply)
   - School
   - Community organization
   - Other ________________________________________
   - Unsure (not clearly stated in article)
5. Grades of youth participants, if listed (select all that apply)
   - 3^rd^ or below
   - 4^th^ grade
   - 5^th^ grade
   - 6^th^ grade
   - 7^th^ grade
   - 8^th^ grade
   - 9^th^ grade
   - 10^th^ grade or above
   - Grades are not stated in the article
6. What city/cities did the curriculum take place in? If cities are not provided, list state(s) or geographic region(s) as described in the article.

____________________________________________________________________

1. What research method(s) was used to evaluate the curriculum in this article? (exclude methods described in referenced previous studies)

____________________________________________________________________

1. How many youth participants were part of the evaluation? ______________________
2. Describe the assessment tool used. (i.e. was it created for this study or a previously used instrument, how many items were used, does the assessment have a name)

____________________________________________________________________

____________________________________________________________________

1. Is the full instrument/all items under study included within the body of the article or in supplemental materials?
   - Yes, in body
   - Yes, in supplemental materials
   - No
2. Does this article include measures of validation and/or reliability of the instrument? If yes, what are the results?
   - No
   - Yes

____________________________________________________________________

____________________________________________________________________

1. What type of outcomes are under study? (Select all that apply)
   - Youth behaviors (use of menstrual products, discussion of health with others, personal hygiene behaviors, etc.)
   - Youth attitudes (gender norms/attitudes, attitudes towards menstruation and body changes)
   - Youth knowledge (knowledge of puberty, menstrual cycle and biological processes, awareness of hygiene and health practices)
   - Youth practices (self-esteem, comfort with body, confidence in managing menstruation, practicing body autonomy)
   - Youth social or emotional wellbeing (emotion regulation, self-efficacy, anxiety, sense of belonging, empathy, etc.)
   - Other youth outcomes ____________________________________________
   - Other outcomes _________________________________________________
2. Summarize the key findings of the article with 3-5 bullets.

____________________________________________________________________

____________________________________________________________________

____________________________________________________________________

____________________________________________________________________

____________________________________________________________________

1. Does the article describe any previous study/testing of the curriculum or the instrument? If yes, briefly describe the findings mentioned in the present article.
   - No
   - Yes

____________________________________________________________________

____________________________________________________________________

1. Reviewer’s summary of how findings related to review questions

___________________________________________________________________________________________________________________________________________________________________________________________________________________________
